# Supplementary material for: Novel Fluorescent Benzimidazole-Hydrazone-Loaded Micellar Carriers for Controlled Release: Impact on Cell Toxicity, Nuclear and Microtubule Alterations in Breast Cancer Cells
Source: Pharmaceutics. 2023 Jun 16;15(6):1753. doi: 10.3390/pharmaceutics15061753 (PMC10302270; doi:10.3390/pharmaceutics15061753)
Supplement: Supplementary file 1 [file pharmaceutics-15-01753-s001.zip › pharmaceutics-2449600-supplementary.pdf]

Supplementary materials

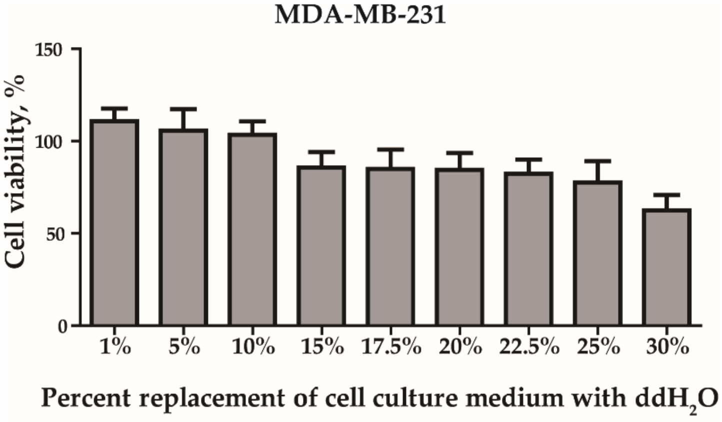

Supplementary Figure S1. Cell viability of MDA-MB-231 cultured for 24 h in cell culture medium replaced with distilled water (ddH_2O). The given percentage corresponds with the cell medium dilution. Cell viability is represented in % and normalized to the control (100% viability), where the cells were grown in 100% cell culture medium.
